# Tissue bridges predict recovery after traumatic and ischemic thoracic spinal cord injury

**DOI:** 10.1212/WNL.0000000000008318

**Published:** 2019-10-15

**Authors:** Dario Pfyffer, Eveline Huber, Reto Sutter, Armin Curt, Patrick Freund

**Affiliations:** From the Spinal Cord Injury Center (D.P., E.H., A.C., P.F.) and Radiology (R.S.), Balgrist University Hospital, Zurich, Switzerland; Wellcome Trust Centre for Neuroimaging (P.F.), UCL Institute of Neurology, University College London, UK; Department of Neurophysics (P.F.), Max Planck Institute for Human Cognitive and Brain Sciences, Leipzig, Germany; and Department of Neurology (P.F.), University Hospital Zurich, Switzerland.

## Abstract

**Objective:**

To investigate the spatiotemporal evolution and predictive properties of intramedullary damage and midsagittal tissue bridges at the epicenter of a thoracic spinal cord injury (SCI) using MRI.

**Methods:**

We retrospectively assessed midsagittal T2-weighted scans from 25 patients with thoracic SCI (14 traumatic, 11 ischemic) at 1 month post-SCI. In 12 patients with SCI, linear mixed-effects models on serial MRI explored temporal trajectories of quantifiable lesion markers (area, length, and width) and tissue bridges. Using partial correlation analysis, we assessed associations between structural lesion characteristics at 1 month post-SCI and recovery at 1 year postinjury, adjusting for baseline clinical status, age, and sex.

**Results:**

Lesion area decreased by 5.68 mm^2^ (*p* = 0.005), lesion length by 2.14 mm (*p* = 0.004), and lesion width by 0.13 mm (*p* = 0.004) per month. Width of tissue bridges increased by 0.06 mm (*p* = 0.019) per month, being similar in traumatic and ischemic SCI (*p* = 0.576). Smaller lesion area, length, width, and wider tissue bridges at 1 month post-SCI predicted better recovery at 1-year follow-up.

**Conclusions:**

Over time, the immediate area of cord damage shrunk while the cystic cavity became demarcated. Adjacent to the cyst, midsagittal tissue bridges became visible. The width of tissue bridges at 1 month post-SCI predicted recovery at 1 year follow-up. Measures of lesion area and tissue bridges early after traumatic and ischemic thoracic SCI therefore allow characterizing the evolution of focal cord damage and are predictive of recovery in thoracic SCI. Thus, lesion extent and tissue bridges hold potential to improve diagnosis and patient stratification in interventional trials.

Spinal cord injury (SCI) leads to persistent physical deficits and significant socio-financial consequences.^1^ SCI results either from a traumatic incidence (e.g., falls) or nontraumatic cause (e.g., ischemia).^2^ Interestingly, independent of lesion etiology, patients with traumatic and ischemic thoracic^3,4^ and cervical^4^ SCI experience a similar clinical recovery. From a pathophysiologic perspective, both etiologies share common neurodegenerative processes, such as neuronal cell death, demyelination, and axonal degeneration.^4^ However, the structural changes underlying thoracic SCI and their prognostic value for clinical recovery are understudied.

MRI is a beneficial tool in clinical diagnostics and prognosis.^5,6^ T2-weighted (T2W) scans from the lesion epicenter have proven useful in tetraplegic patients to quantify the spatiotemporal evolution of the lesion, including intramedullary processes of edema,^7^ hemorrhage,^7^ and spinal cord compression.^7,8^ In addition, it allows determining the extent of midsagittal tissue bridges, their width being a predictor of functional recovery in tetraplegic patients.^6,9^

Despite the lack of studies using neuroimaging biomarkers to predict clinical recovery in paraplegic patients, there is evidence that acute MRI-based lesion characteristics after thoracic SCI relate to the American Spinal Injury Association (ASIA) Impairment Scale (AIS) grade at discharge.^10^ However, in patients with thoracic SCI it remains unknown how the intramedullary damage evolves over time and whether subacute measures of lesion size and midsagittal tissue bridges can be used to predict recovery. Therefore, we planned to assess the spatiotemporal change of lesion extent and investigated associations between the latter at 1 month post-SCI and long-term outcome after traumatic and ischemic thoracic SCI.

## Methods

### Experimental design

In this retrospective study, we included 25 patients with subacute thoracic SCI (14 traumatic and 11 ischemic) who were admitted consecutively to the Balgrist University Hospital (Zurich, Switzerland) between July 2005 and December 2016 (table 1). All patients with traumatic injury underwent surgical decompression. We used data from 12 patients (7 traumatic and 5 ischemic) who had MRI follow-ups for assessing the trajectories of MRI lesion measures within the first 2 years post-SCI. Twenty-one patients with SCI had a baseline MRI scan within the first 2 months post-SCI and clinical assessments at 1 month and 1 year postinjury. These data were used to evaluate associations between subacute lesion measures and clinical outcome measures at follow-up.

**Table 1:**
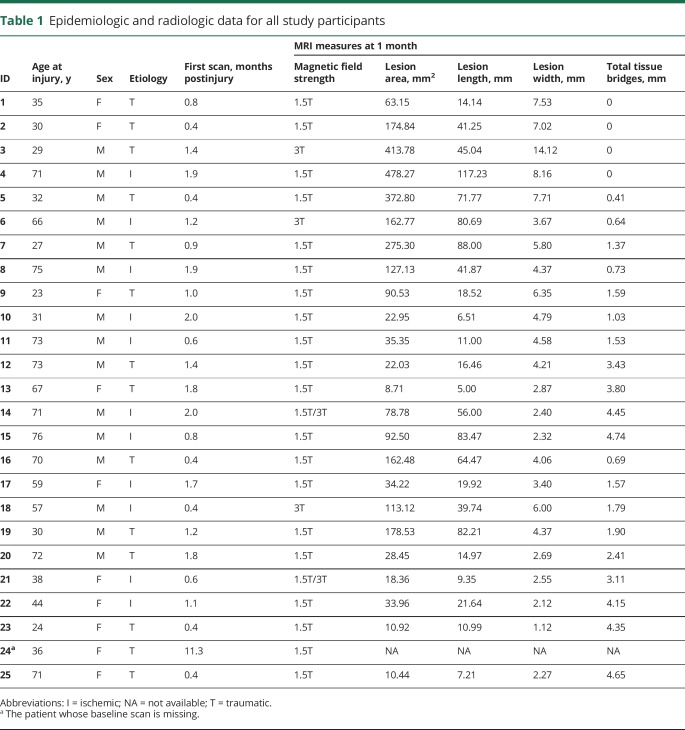
Epidemiologic and radiologic data for all study participants

In this study, we only included patients with a clearly visible lesion on the midsagittal T2W scan. We excluded patients with preexisting neurologic or mental disorders or brain lesions, as well as patients with MRI contraindications.

### Standard protocol approvals, registrations, and patient consents

The local ethics committee approved the study protocol (EK-2010-0271) and all patients with SCI gave informed, written consent prior to study enrollment.

### Clinical assessments

The clinical examination included the lower extremity motor score (LEMS), light touch scores, and pinprick scores of the International Standards for the Neurological Classification of Spinal Cord Injury protocol^11^ (table 2). Using these clinical measures, patients were classified as AIS A (i.e., complete injury), AIS B–D (i.e., incomplete injury), or AIS E (i.e., no functional impairment) according to the ASIA Impairment Scale.

**Table 2:**
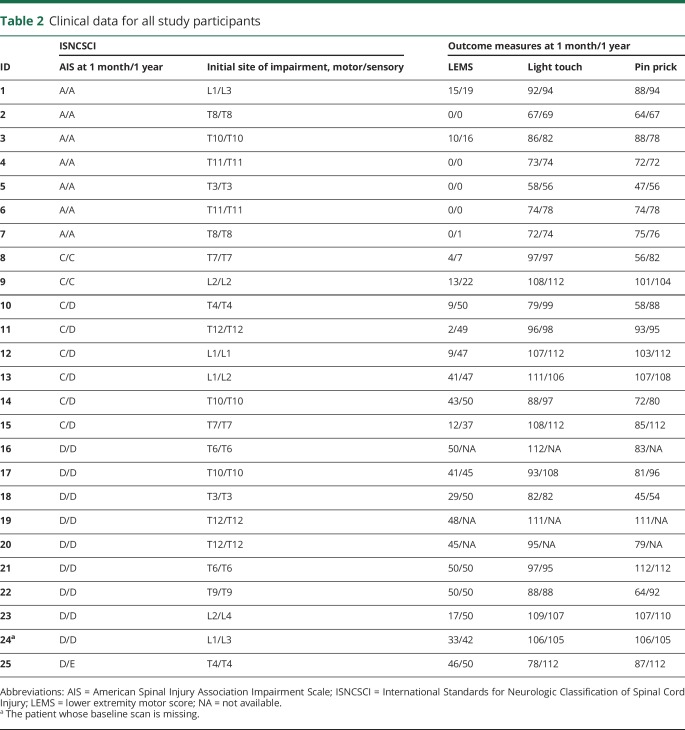
Clinical data for all study participants

### Image acquisition

The MRI protocol consisted of standard sagittal T1-weighted (T1W), sagittal T2W, and axial T2W clinical scans obtained at the lesion site, of which the sagittal T2W scans were the only ones used for analysis. Several field strengths were used (table 1). Of 25 patients, 19 patients were scanned exclusively at 1.5T. Three patients were scanned with a 3T MRI scanner only and 3 patients had both a 1.5T and a 3T MRI scanner during their longitudinal data acquisition. Of the 22 patients scanned with a 1.5T MRI scanner, 17 were scanned with a 1.5T Magnetom Avanto (or the updated Avanto^fit^) scanner, 1 with a 1.5T Magnetom Espree scanner, 1 with a 1.5T Magnetom Symphony scanner (all Siemens Healthcare, Erlangen, Germany), and 1 with a 1.5T Signa HDxt scanner (GE Medical Systems, Waukesha, WI). One patient was scanned with both the Magnetom Avanto and the Magnetom Espree scanner, and 1 patient with both the Magnetom Avanto and the Signa HDxt scanner. Of the 6 patients scanned at 3T, 5 patients were scanned with the 3T Magnetom Skyra (or the updated Skyra^fit^) MRI scanner, and 1 with a 3T Magnetom Verio MRI scanner (both Siemens Healthcare). A 32-channel receive spine coil integrated in the table was used with all scanners. The following values were used for the repetition time (TR), echo time (TE), and flip angle (FA) of the different clinical sequences: sagittal T1W (for 1.5T: TR 542 ms, TE 11 ms, FA 143°; for 3T: TR 553 ms, TE 10 ms, FA 160°), sagittal T2W (for 1.5T: TR 4,082 ms, TE 105 ms, FA 149°; for 3T: TR 4,338 ms, TE 94 ms, FA 156°), and axial T2W (for 1.5T: TR 4,713 ms, TE 105 ms, FA 145°; for 3T: TR 4,698 ms, TE 97 ms, FA 158°). Readout bandwidth was increased to reduce metal artifacts for the different sequences: sagittal T1W and T2W (for 1.5T: 415 Hz/pixel; for 3T: 751 Hz/pixel) and axial T2W (for 1.5T: 330 Hz/pixel; for 3T: 781 Hz/pixel). The spatial resolutions for the 1.5T and 3T scanners were 0.55 × 0.55 × 2.75 mm and 0.57 × 0.57 × 2.75 mm, respectively.

### Image analysis

Intramedullary damage, edema, and hemorrhage manifest as changes of signal intensity on T2W scans. These sagittal T2W scans were considered for the qualitative analysis of edema or hemorrhage by an experienced radiologist (R.S.) prior to lesion identification and characterization. MRI scans with insufficient image quality or lesion visibility due to metal artifacts were excluded.

**Figure 1:**
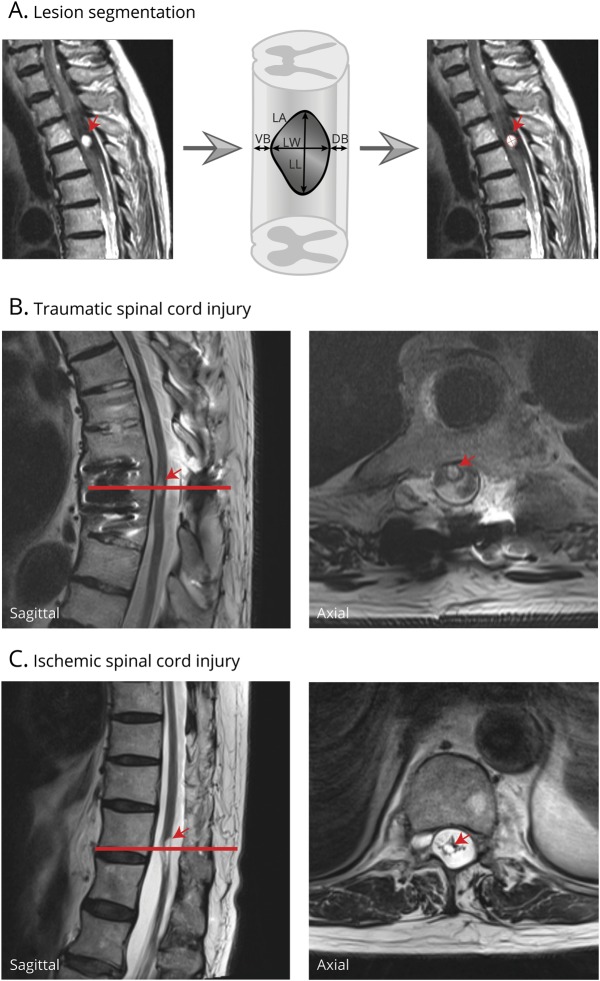
T2-weighted (T2W) midsagittal MRI slices at the thoracic lesion site (A) Schematic lesion segmentation including a typical T2W midsagittal slice, which is overlaid with a schematic drawing of the quantitative MRI measures analyzed (lesion area [LA], lesion length [LL], lesion width [LW], ventral midsagittal tissue bridges [VB], and dorsal midsagittal tissue bridges [DB]). (B, C) Representative sagittal and axial images of a patient with traumatic SCI (B) and a patient with ischemic SCI (C) in the chronic phase. The arrows mark the intramedullary cystic cavity.

Two raters (D.P. and E.H.) were blinded to patient identity and scan time point prior to segmentation. The midsagittal slice of all sagittal T2W slices was identified for every patient and scan time point. We used Jim 7.0 software (Xinapse Systems, Aldwincle, UK) to quantitatively assess the total midsagittal lesion area, rostro-caudal lesion length, anterior-posterior lesion width, and the total width of tissue bridges (sum of ventral and dorsal tissue bridges) as imaging measurers on the midsagittal T2W images for all time points (figure 1A).^6^ The raters defined spared tissue bridges (likely including not only functional but also necrotic and glial scar tissue) within the spinal cord as the hypointense regions between the relatively hyperintense regions representing the intramedullary lesion cavity on one side and the CSF on the other side. Figure 1, B and C, shows sagittal (first column) and axial (second column) images of a patient with chronic traumatic and chronic ischemic SCI. The cystic cavities are marked by the red arrows.

Intraobserver and interobserver reliability were calculated for all imaging measures in 7 randomly selected scans. The corresponding coefficients of variation (COV) were 5.3% for the intraobserver reliability and 7.0% for the interobserver reliability, which were a bit higher compared to the intraobserver and interobserver COV (4.3% and 5.2%, respectively) of the Huber et al.^6^ study with tetraplegic patients.

### Statistical analysis

We used Stata software (version 14; StataCorp LP, College Station, TX) and paired 1-tailed *t* tests to investigate the functional recovery (i.e., light touch score, pinprick score, and LEMS) over 1 year postinjury. We applied a one-way analysis of variance followed by a Bonferroni post hoc test for pairwise comparison of AIS A, AIS C, and AIS D patients regarding their structural imaging characteristics (i.e., lesion area, lesion length, lesion width, and width of midsagittal tissue bridges) at baseline. Unpaired 2-tailed *t* tests were used to compare these lesion characteristics at baseline between patients with traumatic and ischemic SCI.

Linear mixed-effects models were used to calculate the rates of change over time for the lesion area (n = 12), lesion length (n = 12), lesion width (n = 12), and the width of midsagittal tissue bridges (n = 11) and to investigate differences in time course of MRI changes between patients with traumatic and ischemic paraplegic SCI. Age and sex were included as a fixed effect and between-patient variability and time since injury as random effects. *p* Values are reported in the Results and in table 3.

**Table 3:**
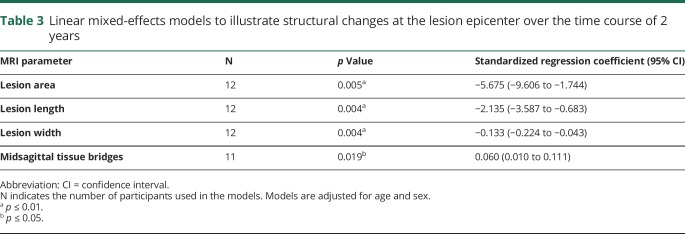
Linear mixed-effects models to illustrate structural changes at the lesion epicenter over the time course of 2 years

We used partial correlation analysis to investigate the associations between quantitative structural characteristics at 1 month postinjury and functional recovery at 1 year follow-up (n = 21). Lesion measures of all patients were used in the model to describe the associations between imaging measures (i.e., lesion area, lesion length, lesion width, and width of tissue bridges) and clinical outcomes (i.e., LEMS, light touch score, and pinprick score). The regression models were adjusted for age, sex, and clinical baseline scores (i.e., 1 month) and the potential confounders were only retained if the covariates were significant or if they had a substantial effect on the partial correlation coefficient of interest. These coefficients from the regression analysis and the *p* values are reported in the Results and in table 4.

**Table 4:**
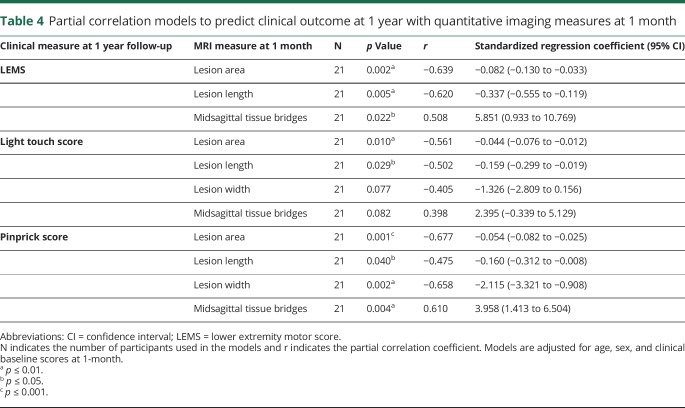
Partial correlation models to predict clinical outcome at 1 year with quantitative imaging measures at 1 month

Results were regarded as significant when the *p* values were smaller than 0.05. Only significant results are reported and nonsignificant results with a trend (*p* < 0.1) are discussed.

### Data availability

Anonymized grouped data will be shared by request from a qualified investigator.

## Results

### Clinical, epidemiologic, and radiologic characteristics

Twenty-five patients (14 traumatic and 11 ischemic) had thoracic SCI (15 men, age [mean ± SD] 51.20 ± 20.07 years). The time interval between injury and baseline scan (i.e., 1-month scan) was 33.21 ± 17.56 days. However, 1 patient did not have a baseline scan (indicated by ^a^ in tables 1 and 2). During the course of the first year post-SCI, patients recovered by 4 ± 9 points (from 90 to 94 points) on the light touch score (*p* = 0.023, n = 22), by 9 ± 11 points (from 81 to 90 points) on the pinprick score (*p* < 0.001, n = 22), and by 12 ± 15 points (from 19 to 31) on the LEMS (*p* < 0.001, n = 22). Notably, clinical data at 1 year post-SCI were missing for 3 patients (table 2).

Seven patients were classified as AIS A and 18 as AIS C or D at baseline. At 1 year post-SCI, the same 7 patients were classified as AIS A, 6 patients improved from AIS C to AIS D, and 1 patient from AIS D to AIS E (table 2). At 1 month postinjury, AIS A patients (n = 7) showed a lesion area of 277.3 ± 151.4 mm^2^, a lesion length of 65.4 ± 34.4 mm, a lesion width of 7.7 ± 3.2 mm, and a width of midsagittal tissue bridges of 0.3 ± 0.5 mm. AIS C (n = 8) and AIS D (n = 9) patients had a lesion area of 59.7 ± 42.9 mm^2^ and 65.6 ± 67.1 mm^2^, a lesion length of 29.9 ± 28.1 mm and 30.1 ± 26.7 mm, a lesion width of 4.0 ± 1.4 mm and 3.2 ± 1.5 mm, and a width of tissue bridges of 2.7 ± 1.6 mm and 2.7 ± 1.4 mm, respectively. There was an overall difference in lesion area (*p* < 0.001), lesion length (*p* = 0.046), lesion width (*p* < 0.001), and width of midsagittal tissue bridges (*p* = 0.002) at baseline among AIS A, AIS C, and AIS D patients. Pairwise subgroup comparisons showed that AIS A patients had a greater lesion area (AIS A vs C: *p* = 0.001, AIS A vs D: *p* = 0.001), lesion length by trend (AIS A vs C: *p* = 0.090, AIS A vs D: *p* = 0.081), lesion width (AIS A vs C: *p* = 0.007, AIS A vs D: *p* = 0.001), and a smaller width of midsagittal tissue bridges (AIS A vs C: *p* = 0.007, AIS A vs D: *p* = 0.005) at 1 month post-SCI compared to AIS C and D patients. However, for none of the imaging measures was there a difference between AIS C and AIS D patients (lesion area: *p* = 1.000, lesion length: *p* = 1.000, lesion width: *p* = 1.000, tissue bridges: *p* = 1.000) at that time point. At 1 month postinjury, there was no difference in lesion area (traumatic: 139.4 ± 140.3 mm^2^, ischemic: 108.9 ± 131.5 mm^2^, *p* = 0.590), lesion length (traumatic: 36.9 ± 30.3 mm, ischemic: 44.3 ± 36.3 mm, *p* = 0.593), lesion width (traumatic: 5.4 ± 3.4 mm, ischemic: 4.0 ± 1.8 mm, *p* = 0.244), or width of tissue bridges (traumatic: 1.9 ± 1.7 mm, ischemic: 2.2 ± 1.7 mm, *p* = 0.704) between patients with traumatic and ischemic SCI.

Twenty of 24 patients had midsagittal tissue bridges with an average width of 2.0 ± 1.7 mm at 1 month post-SCI. From 7 AIS A patients, 4 had no tissue bridges at 1 month, and 3 had residual tissue bridges at that time point (table 1). Minor edema was found in 7 patients and minor hemorrhage in 1 patient.

### Time course of lesion characteristic changes at the focal injury site

**Figure 2:**
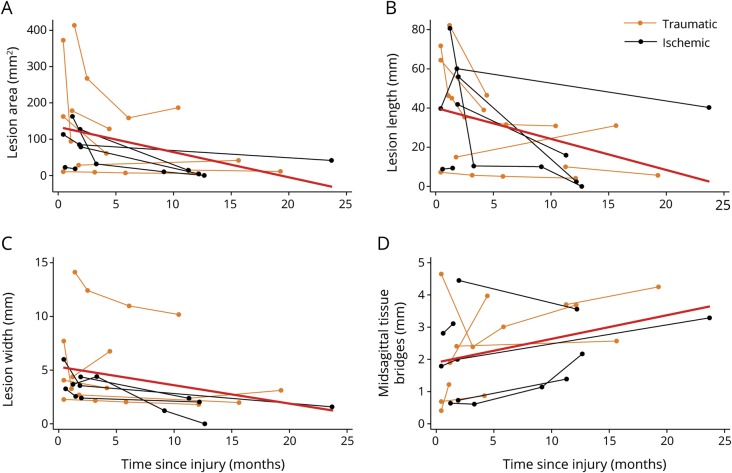
Spatiotemporal changes of the lesion at the focal injury site MRI changes over time are illustrated for lesion area (A), lesion length (B), lesion width (C), and width of midsagittal tissue bridges (D). Patients with traumatic injury are depicted in orange and patients with ischemic injury in black. The red line indicates the linear fit of the mixed-effects model.

In the 12 patients with SCI who had longitudinal follow-up scans, lesion area decreased by 5.68 mm^2^ per month (*p* = 0.005, n = 12, 95% confidence interval [CI] −9.606 to 1.744 mm^2^), lesion length declined by 2.14 mm per month (*p* = 0.004, n = 12, 95% CI −3.587 to 0.683 mm), lesion width decreased by 0.13 mm per month (*p* = 0.004, n = 12, 95% CI −0.224 to 0.043 mm), and the width of midsagittal tissue bridges increased by 0.06 mm per month (*p* = 0.019, n = 11, 95% CI 0.010 to 0.111 mm) (figure 2 and table 3). The spatiotemporal evolution of the MRI measures did not differ between the traumatic and ischemic patient group (lesion area: *p* = 0.318, lesion length: *p* = 0.863, lesion width: *p* = 0.683, width of midsagittal tissue bridges: *p* = 0.576).

### Associations between lesion extent at 1 month and clinical recovery at 1 year

**Figure 3:**
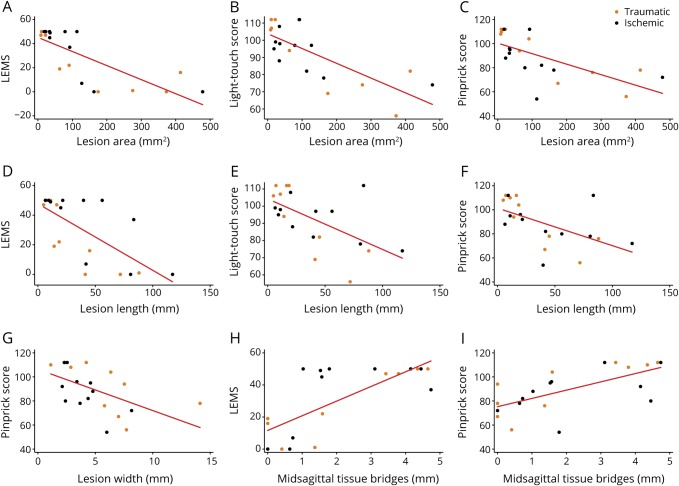
Associations between quantitative structural characteristics at 1 month and functional recovery at 1 year (A–I) Correlations between baseline MRI measures and clinical outcome measures at 1-year follow-up. Lesion area (A–C) and lesion length (D–F) are negatively associated with lower extremity motor score (LEMS) (A, D), light touch score (B, E), and pinprick score (C, F). Lesion width negatively correlates with pinprick score (G) and midsagittal tissue bridges positively correlate with LEMS (H) and pinprick score (I). Patients with traumatic injury are depicted in orange and patients with ischemic injury in black. The red line indicates the linear fit of the partial correlation analysis.

Wider midsagittal tissue bridges at 1 month post-SCI were associated with better LEMS (*p* = 0.022, n = 21, *r* = 0.508) and pinprick scores (*p* = 0.004, n = 21, *r* = 0.610) at 1 year postinjury. Furthermore, wider midsagittal tissue bridges were associated by trend with better light touch scores (*p* = 0.082, n = 21, *r* = 0.398). Smaller lesion area and lesion length at 1 month were associated with better LEMS (lesion area: *p* = 0.002, n = 21, *r* = −0.639; lesion length: *p* = 0.005, n = 21, *r* = −0.620), light touch scores (lesion area: *p* = 0.010, n = 21, *r* = −0.561; lesion length: *p* = 0.029, n = 21, *r* = −0.502), and pinprick scores (lesion area: *p* = 0.001, n = 21, *r* = −0.677; lesion length: *p* = 0.040, n = 21, *r* = −0.475) at 1 year post-SCI. Smaller lesion width at 1 month was related to better pinprick scores (*p* = 0.002, n = 21, *r* = −0.658) and by trend to better light touch scores (*p* = 0.077, n = 21, *r* = −0.405) at 1 year (figure 3 and table 4).

## Discussion

This study is the first showing the spatiotemporal evolution of intramedullary damage and the associations between structural lesion characteristics and clinical recovery in patients with subacute thoracic SCI. While the lesion size decreased over time, subtle increases in the size of midsagittal tissue bridges paralleled patients' recovery. Specifically, smaller lesion size and greater width of midsagittal tissue bridges at 1 month post-SCI predicted better long-term clinical recovery. Interestingly, over time intramedullary lesion changes are similar between patients with traumatic and ischemic SCI.

### Time course of lesion characteristic changes at the focal injury site

In the 12 patients with serial follow-up scans, lesion area, lesion length, and lesion width decreased over time. The observed time course of these MRI measure changes at the focal injury site could be attributed to a removal of myelin debris and axonal fragments.^6,12^ Midsagittal tissue bridges, on the other hand, showed signs of recovery in terms of increasing width over time. However, the increasing width of tissue bridges might be caused by a better imaging resolution and therefore more exact measures of tissue bridges over time (i.e., follow-up scan with 3T compared to baseline scan with 1.5T) in some of the patients. In addition, there is a small chance that the lesions are slightly overestimated and the tissue bridges underestimated at 1 month postinjury as we cannot be completely sure that hemorrhage and edema are fully resolved in those patients with a relatively early baseline scan. Nevertheless, remaining signs of hemorrhage and edema were small and in general, lesion borders were clearly identifiable, thus allowing a precise characterization of the lesion extent at 1 month post-SCI and of structural changes over time.^6,13^

Interestingly, for none of the imaging measures at the lesion epicenter was there a significant difference at baseline and in change over time between the traumatic and the ischemic patient group. The etiology in this study cohort thus does not seem to have a substantial influence on the disease course and the neurologic recovery trajectory.^4^ These results are supported by previous studies, in which patients with traumatic and ischemic SCI were shown to have similar neurologic deficits, rates of recovery, and rehabilitative potential.^3,4^ These analogies are likely driven by similar underlying molecular and structural changes postinjury that traumatic and ischemic lesions have in common.^4^ At the epicenter of the lesion, these include cell death, hemorrhage, inflammation, ischemic dysfunction, and oxidative stress,^14^ consequently leading to axonal and neuronal deficit and ultimately resulting in the formation of a cystic cavity.^15–18^

With respect to the lesion level, our finding of increasing midsagittal tissue bridges over time is in contrast to what has been observed in cervical SCI,^6^ where the size of tissue bridges remained unchanged during the first year after injury. However, in the latter study, only patients with traumatic SCI and different scanning time points were used as structural changes up to 1 year after injury were investigated in comparison to 2 years and the mixture of patients with traumatic and ischemic injury in this study. Furthermore, the MRI signal-to-noise ratio is better within the cervical cord (e.g., lower artifacts due to metal implants). This makes it difficult to compare the temporal evolution of the imaging measures between cervical^6^ and thoracic SCI. In addition, patients with tetraplegic and paraplegic SCI vary in terms of different anatomical (e.g., gray to white matter ratio) and functional properties of the cervical and the thoracic spinal cord.^19–21^ The injury courses and therefore also the temporal changes of the lesion characteristics as well as the treatment response and the prediction of neurologic outcome of these 2 patient groups are thus likely to differ.^22^ In fact, thoracic injuries were shown to have specific mechanical and physiologic properties because of a distinctive vascular supply and energy effect needed for an injury appearance, which are different from cervical injuries.^23,24^ So far, to our knowledge, no studies have shown differences in postinjury interventions and treatments depending on the neurologic level of injury. However, our findings highlight the importance and necessity to further investigate the microstructural and macrostructural changes underlying thoracic lesions for a better understanding of the similarities and differences between cervical and thoracic SCI regarding the pathology, therapeutic treatment, and clinical outcome.

### Associations between lesion extent at 1 month and clinical recovery at 1 year

Prediction of individual trajectories of functional recovery after SCI is challenging due to its molecular and structural heterogeneity^25^ and due to the limited prognostic value of lesion level and severity assessment,^26,27^ clinical examinations (for example, ASIA scores),^3^ and electrophysiologic measurements.^3,28^ Crucially, neuroimaging biomarkers of lesion size and tissue bridges at 1 month post-SCI were predictive of clinical outcome at 1 year follow-up, independent of baseline clinical status. In general, the width of midsagittal tissue bridges was higher with less severe AIS grade (i.e., more severely injured patients had a greater lesion size and smaller midsagittal tissue bridges) at 1 month after the injury and this is in agreement with tetraplegic patients.^6^ Specifically, at 1 month post-SCI, AIS A patients showed a significantly greater lesion extent and a smaller width of tissue bridges than AIS C and D patients. However, patients with an AIS grade C did not differ from patients with an AIS grade D. This is what we expected, as both AIS C and D patients are motor incomplete with preserved motor function below the neurologic level.^11^ Interestingly, the level of remaining muscle function in motor incomplete thoracic SCI thus seems not to be profoundly determined by the lesion extent visible on MRI. However, a potential correlation might also be confounded by imaging inaccuracies (e.g., resolution, signal-to-noise ratio) or parasagittal tissue bridges. The age- and sex-independency of the size and location of tissue bridges suggests that the injury mechanisms are more prominent determinants of spared tissue than the demographics of the patients with SCI. Interestingly, 3 out of 7 AIS A patients had midsagittal tissue bridges at 1 month postinjury. However, this might be necrotic or glial scar tissue and not represent spared tissue including functional fibers. Further motor and sensory electrophysiologic studies need to determine the functional role of spared tissue adjacent to the intramedullary damage.

Huber et al.^6^ tested the potential functionality of spared afferent and efferent fibers after SCI in tetraplegic patients. They reported a preserved information flow using electrophysiologic recordings and observed associations between subacute lesion measures and clinical outcome at 1 year. In addition, Hupp et al.^29^ depicted electrophysiologic measures (e.g., motor and sensory evoked potentials) as clinical predictors of functional recovery in patients with traumatic cervical SCI. These findings are linked to the relations of smaller lesion size and larger width of tissue bridges to better long-term clinical recovery reported for patients with thoracic SCI in this study. The observed recovery of these patients with thoracic SCI is likely mediated by spared white matter fibers and dependent on the location and extent thereof.^27,30,31^ On the other hand, tract specificity and size of the lesion determine the type and magnitude of clinical impairment and just recently, lateral corticospinal tract damage has been shown to correlate with motor output in patients with incomplete SCI.^32^ As smaller lesions probably lead to less neuronal damage and axonal degeneration in the spinal cord, fewer fibers of upper or lower motoneurons are likely to be affected,^33^ which might keep the compensatory potential on a higher level. Spared motor or sensory pathways, even silent ones (i.e., no clinical evidence of fiber tract function) in clinically complete SCI,^34^ may contribute to improved treatment-induced^35^ or spontaneous^36,37^ recovery in chronic SCI. A prominent hypothesis is that the recovery process might be driven by underlying plastic adaptations and changes of intact fibers after the injury.^38^ Preclinical studies indeed showed that such SCI-induced changes can be observed within the reticulospinal tract,^39,40^ the corticospinal tract,^31,39,41^ and intraspinal circuits^42^ and are associated with functional recovery.^31,39,40^ Spontaneous repair of damaged spinal tracts, on the other hand, is limited in animals^43^ and nearly absent in humans.^38^

By which mechanisms the observed recovery of the patients in our study is driven remains unanswered. However, the reported relation of smaller lesion size and larger width of tissue bridges to better long-term clinical recovery suggests functionally intact tract fibers as a mediator of recovery, the magnitude of the latter being dependent on the lesion extent.

The current study has some limitations. First, this is a retrospective monocentric study with specific inclusion criteria, which may have led to a selection bias. Even though this produced a homogeneous dataset, it may not reflect the general SCI population. In addition, men and women were not equally represented in our patient group. However, male and female patients of the general SCI population are not equally distributed either, with an actual male/female ratio of 4:1.^44^ Second, in comparison to Huber et al.,^6^ the intraobserver (5.3% vs 4.3%) and interobserver (7.0% vs 5.2%) COV were slightly higher. Nevertheless, they are notably low and therefore reflect an accurate and reliable method of manual lesion segmentation. Third, assessment of tissue bridges on T2W axial slices was not possible due to the low spatial resolution. Instead, the lesion size and tissue bridges could be investigated in the midsagittal plane and even in the presence of metal artifacts near the lesion site. This does not include parasagittal lesion parts and does not reflect the 3D shape of the damage. However, this would be necessary for precise correlations between specific tracts and the corresponding functions (i.e., descending motor tracts and ascending sensory tracts) and should, together with a separate analysis of ventral and dorsal tissue bridges, be addressed in future studies. In addition, functional electrophysiologic assessments could complement the imaging and clinical information about lesion completeness and sparing of fiber tracts. Note that there was no baseline scan for one patient and clinical data at 1-year follow-up was not available for 3 patients, reducing the number of patients used for outcome prediction from 25 to 21. Finally, the sample sizes of patients with traumatic and ischemic injury should be increased in future studies investigating the neurologic and functional similarities of these 2 SCI patient groups differing in etiology. We next aim to embark on multicenter studies to validate our findings in a greater cohort. Further steps to increase the prognostic value could be stratification of patients according to their AIS grade or lesion level, follow-up scans during the acute and subacute stage after injury,^8^ and the investigation of macrostructural and microstructural changes remote from the injury with advanced quantitative MRI.^45^

This study shows the spatiotemporal dynamics of intramedullary damage in subacute thoracic SCI using longitudinal MRI assessments during the first 2 years after SCI. We show that analysis of structural lesion characteristics acquired at the lesion epicenter using conventional MRI in thoracic SCI in the subacute phase provides a basis to compare the natural evolution of intramedullary lesion changes between patients with traumatic and ischemic injury. Moreover, the assessment of midsagittal tissue bridges reliably predicts functional recovery after traumatic and nontraumatic SCI. The measures of lesion severity and tissue preservation early after thoracic injury furthermore hold promise to be implicated as accurate and reliable neuroimaging biomarkers for the diagnostic workup and patient stratification for both subacute and chronic clinical trials.

## Glossary

AIS: American Spinal Injury Association Impairment Scale
ASIA: American Spinal Injury Association
CI: confidence interval
COV: coefficient of variation
FA: flip angle
LEMS: lower extremity motor score
SCI: spinal cord injury
T1W: T1-weighted
T2W: T2-weighted
TE: echo time
TR: repetition time

## References

[1] Ahuja CS, Nori S, Tetreault L, et al. Traumatic spinal cord injury: repair and regeneration. Neurosurgery 2017;80:S9–S22. 28350947 10.1093/neuros/nyw080

[2] Noonan VK, Thorogood NP, Fingas M, et al. The validity of administrative data to classify patients with spinal column and cord injuries. J Neurotrauma 2013;30:173–180. 23002989 10.1089/neu.2012.2441

[3] Iseli E, Cavigelli A, Dietz V, Curt A. Prognosis and recovery in ischaemic and traumatic spinal cord injury: clinical and electrophysiological evaluation. J Neurol Neurosurg Psychiatry 1999;67:567–571. 10519858 10.1136/jnnp.67.5.567 PMC1736605

[4] Scivoletto G, Laurenza L, Mammone A, Foti C, Molinari M. Recovery following ischemic myelopathies and traumatic spinal cord lesions. Spinal Cord 2011;49:897–902. 21468041 10.1038/sc.2011.31

[5] Boldin C, Raith J, Fankhauser F, Haunschmid C, Schwantzer G, Schweighofer F. Predicting neurologic recovery in cervical spinal cord injury with postoperative MR imaging. Spine 2006;31:554–559. 16508551 10.1097/01.brs.0000201274.59427.a4

[6] Huber E, Lachappelle P, Sutter R, Curt A, Freund P. Are midsagittal tissue bridges predictive of outcome after cervical spinal cord injury? Ann Neurol 2017;81:740–748. 28393423 10.1002/ana.24932

[7] Miyanji F, Furlan JC, Aarabi B, Arnold PM, Fehlings MG. Acute cervical traumatic spinal cord injury: MR imaging findings correlated with neurologic outcome: prospective study with 100 consecutive patients. Radiology 2007;243:820–827. 17431129 10.1148/radiol.2433060583

[8] Farhadi HF, Kukreja S, Minnema A, et al. Impact of admission imaging findings on neurological outcomes in acute cervical traumatic spinal cord injury. J Neurotrauma 2018;35:1398–1406. 29361876 10.1089/neu.2017.5510 PMC13175224

[9] O'Dell DR, Weber KA, Berliner JC, et al. Midsagittal tissue bridges are associated with walking ability in incomplete spinal cord injury: a magnetic resonance imaging case series. J Spinal Cord Med 2018:1–4. 10.1080/10790268.2018.1527079 PMC7054908 30346248

[10] Mabray MC, Talbott JF, Whetstone WD, et al. Multidimensional analysis of magnetic resonance imaging predicts early impairment in thoracic and thoracolumbar spinal cord injury. J Neurotrauma 2016;33:954–962. 26414451 10.1089/neu.2015.4093 PMC4876497

[11] Kirshblum SC, Burns SP, Biering-Sorensen F, et al. International standards for neurological classification of spinal cord injury (revised 2011). J Spinal Cord Med 2011;34:535–546. 22330108 10.1179/204577211X13207446293695 PMC3232636

[12] Noble LJ, Wrathall JR. Spinal cord contusion in the rat: morphometric analyses of alterations in the spinal cord. Exp Neurol 1985;88:135–149. 3979507 10.1016/0014-4886(85)90119-0

[13] Shimada K, Tokioka T. Sequential MR studies of cervical cord injury: correlation with neurological damage and clinical outcome. Spinal Cord 1999;37:410–415. 10432260 10.1038/sj.sc.3100858

[14] Anwar MA, Al Shehabi TS, Eid AH. Inflammogenesis of secondary spinal cord injury. Front Cel Neurosci 2016;10:98. 10.3389/fncel.2016.00098 PMC4829593 27147970

[15] Wilson JR, Forgione N, Fehlings MG. Emerging therapies for acute traumatic spinal cord injury. CMAJ 2013;185:485–492. 23228995 10.1503/cmaj.121206 PMC3612151

[16] Zhang C, Chen K, Han X, et al. Diffusion tensor imaging in diagnosis of post-traumatic syringomyelia in spinal cord injury in rats. Med Sci Monit 2018;24:177–182. 29311540 10.12659/MSM.907955 PMC5771161

[17] Krebs J, Koch HG, Hartmann K, Frotzler A. The characteristics of posttraumatic syringomyelia. Spinal Cord 2016;54:463–466. 26620880 10.1038/sc.2015.218

[18] Fassbender JM, Whittemore SR, Hagg T. Targeting microvasculature for neuroprotection after. SCI Neurotherapeutics 2011;8:240–251. 21360237 10.1007/s13311-011-0029-1 PMC3101824

[19] Harrop JS, Maltenfort MG, Geisler FH, et al. Traumatic thoracic ASIA A examinations and potential for clinical trials. Spine 2009;34:2525–2529. 19927102 10.1097/BRS.0b013e3181bd1402

[20] Kingwell SP, Noonan VK, Fisher CG, et al. Relationship of neural axis level of injury to motor recovery and health-related quality of life in patients with a thoracolumbar spinal injury. J Bone Joint Surg Am 2010;92:1591–1599. 20595564 10.2106/JBJS.I.00512

[21] Solsberg MD, Lemaire C, Resch L, Potts DG. High-resolution MR imaging of the cadaveric human spinal cord: normal anatomy. AJNR Am J Neuroradiol 1990;11:3–7. 2105614 PMC8332496

[22] Wilson JR, Jaja BNR, Kwon BK, et al. Natural history, predictors of outcome, and effects of treatment in thoracic spinal cord injury: a multi-center cohort study from the North American Clinical Trials Network. J Neurotrauma 2018;35:2554–2560. 29665733 10.1089/neu.2017.5535

[23] Koizumi M, Ueda Y, Iida J, et al. Upper thoracic spinal cord injury without vertebral bony lesion: a report of two cases. Spine 2002;27:E467–E470. 12438998 10.1097/00007632-200211010-00020

[24] el-Khoury GY, Whitten CG. Trauma to the upper thoracic spine: anatomy, biomechanics, and unique imaging features. AJR Am J Roentgenol 1993;160:95–102. 8416656 10.2214/ajr.160.1.8416656

[25] Zorner B, Blanckenhorn WU, Dietz V, EM-SCI Study Group, Curt A. Clinical algorithm for improved prediction of ambulation and patient stratification after incomplete spinal cord injury. J Neurotrauma 2010;27:241–252. 19645527 10.1089/neu.2009.0901

[26] Scivoletto G, Tamburella F, Laurenza L, Torre M, Molinari M. Who is going to walk? A review of the factors influencing walking recovery after spinal cord injury. Front Hum Neurosci 2014;8:141. 24659962 10.3389/fnhum.2014.00141 PMC3952432

[27] Fehlings MG, Tator CH. The relationships among the severity of spinal cord injury, residual neurological function, axon counts, and counts of retrogradely labeled neurons after experimental spinal cord injury. Exp Neurol 1995;132:220–228. 7789460 10.1016/0014-4886(95)90027-6

[28] Curt A, Keck ME, Dietz V. Functional outcome following spinal cord injury: significance of motor-evoked potentials and ASIA scores. Arch Phys Med Rehabil 1998;79:81–86. 9440423 10.1016/s0003-9993(98)90213-1

[29] Hupp M, Pavese C, Bachmann LM, Koller R, Schubert M, Group ES. Electrophysiological multimodal assessments improve outcome prediction in traumatic cervical spinal cord injury. J Neurotrauma 2018;35:2916–2923. 29792368 10.1089/neu.2017.5576

[30] You SW, Chen BY, Liu HL, et al. Spontaneous recovery of locomotion induced by remaining fibers after spinal cord transection in adult rats. Restor Neurol Neurosci 2003;21:39–45. 12808201

[31] Rosenzweig ES, Courtine G, Jindrich DL, et al. Extensive spontaneous plasticity of corticospinal projections after primate spinal cord injury. Nat Neurosci 2010;13:1505–1510. 21076427 10.1038/nn.2691 PMC3144760

[32] Smith AC, Weber KA II, O'Dell DR, Parrish TB, Wasielewski M, Elliott JM. Lateral corticospinal tract damage correlates with motor output in incomplete spinal cord injury. Arch Phys Med Rehabil 2018;99:660–666. 29107041 10.1016/j.apmr.2017.10.002 PMC5871547

[33] Grumbles RM, Thomas CK. Motoneuron death after human spinal cord injury. J Neurotrauma 2017;34:581–590. 27349409 10.1089/neu.2015.4374 PMC5286554

[34] Wrigley PJ, Siddall PJ, Gustin SM. New evidence for preserved somatosensory pathways in complete spinal cord injury: a fMRI study. Hum Brain Mapp 2018;39:588–598. 29080262 10.1002/hbm.23868 PMC6866574

[35] Angeli CA, Boakye M, Morton RA, et al. Recovery of over-ground walking after chronic motor complete spinal cord injury. N Engl J Med 2018;379:1244–1250. 30247091 10.1056/NEJMoa1803588

[36] Kirshblum S, Millis S, McKinley W, Tulsky D. Late neurologic recovery after traumatic spinal cord injury. Arch Phys Med Rehabil 2004;85:1811–1817. 15520976 10.1016/j.apmr.2004.03.015

[37] Choe AS, Belegu V, Yoshida S, et al. Extensive neurological recovery from a complete spinal cord injury: a case report and hypothesis on the role of cortical plasticity. Front Hum Neurosci 2013;7:290. 23805087 10.3389/fnhum.2013.00290 PMC3691521

[38] Curt A, Van Hedel HJ, Klaus D, Dietz V, EM-SCI Study Group. Recovery from a spinal cord injury: significance of compensation, neural plasticity, and repair. J Neurotrauma 2008;25:677–685. 18578636 10.1089/neu.2007.0468

[39] Courtine G, Song B, Roy RR, et al. Recovery of supraspinal control of stepping via indirect propriospinal relay connections after spinal cord injury. Nat Med 2008;14:69–74. 18157143 10.1038/nm1682 PMC2916740

[40] Asboth L, Friedli L, Beauparlant J, et al. Cortico-reticulo-spinal circuit reorganization enables functional recovery after severe spinal cord contusion. Nat Neurosci 2018;21:576–588. 29556028 10.1038/s41593-018-0093-5

[41] Ghosh A, Haiss F, Sydekum E, et al. Rewiring of hindlimb corticospinal neurons after spinal cord injury. Nat Neurosci 2010;13:97–104. 20010824 10.1038/nn.2448

[42] Courtine G, Gerasimenko Y, van den Brand R, et al. Transformation of nonfunctional spinal circuits into functional states after the loss of brain input. Nat Neurosci 2009;12:1333–1342. 19767747 10.1038/nn.2401 PMC2828944

[43] Nashmi R, Imamura H, Tator CH, Fehlings MG. Serial recording of somatosensory and myoelectric motor evoked potentials: role in assessing functional recovery after graded spinal cord injury in the rat. J Neurotrauma 1997;14:151–159. 9104932 10.1089/neu.1997.14.151

[44] Jackson AB, Dijkers M, Devivo MJ, Poczatek RB. A demographic profile of new traumatic spinal cord injuries: change and stability over 30 years. Arch Phys Med Rehabil 2004;85:1740–1748. 15520968 10.1016/j.apmr.2004.04.035

[45] Freund P, Seif M, Weiskopf N, et al. MRI in traumatic spinal cord injury: from clinical assessment to neuroimaging biomarkers. Lancet Neurol Epub Aug 9, 2019. pii: S1474-4422(19)30138-3. 10.1016/S1474-4422(19)30138-3 31405713

